# NOD/scid IL‐2Rγ^null^ mice reconstituted with peripheral blood mononuclear cells from patients with Crohn's disease reflect the human pathological phenotype

**DOI:** 10.1002/iid3.516

**Published:** 2021-09-09

**Authors:** Anna‐Lena Unterweger, Alena Rüscher, Marietta Seuß, Paula Winkelmann, Florian Beigel, Leandra Koletzko, Simone Breiteneicher, Matthias Siebeck, Roswitha Gropp, Attila Aszodi

**Affiliations:** ^1^ Department of General, Visceral and Transplantation Surgery Hospital of the LMU Munich Germany; ^2^ Department of Medicine II Hospital of the LMU, Munich München Germany; ^3^ Department of Experimental Surgery and Regenerative Medicine Hospital of the LMU Planegg Germany

**Keywords:** colitis ulcerosa, Crohn's disease, fibrosis, NOD/Scid IL‐2Rγ^null^

## Abstract

**Introduction:**

Crohn's disease (CD) is characterized by pronounced intestinal fibrosis and severe mucosal damage and conventional animal models are limited to reflect these pathological manifestations. The aim of this study was to examine whether the combination of patient immune‐profiling and preclinical studies in a mouse model based on NOD/scid IL‐2Rγ^null^ (NSG) reconstituted with peripheral blood mononuclear cells (PBMC) from CD patients has the capacity to harmonize ex vivo human and in vivo animal studies.

**Methods:**

Immunological profiles of CD (*n* = 24) and ulcerative colitis (UC) patients (*n* = 47) were established by flow cytometry of subgroups of immune cells and subjected to hierarchical cluster and estimation graphics analyses. Pathological phenotypes of NSG mice, which were reconstituted with PBMC from CD, UC, and non‐IBD donors (NSG‐CD, NSG‐UC, and NSG‐non‐IBD) were compared. Readouts were the clinical, colon, and histological scores; subtypes of immune cells from spleen and colon; and levels of inflammatory markers, such as c‐reactive protein (CRP), monocyte chemotactic protein (MCP)‐3, transforming growth factor‐beta (TGFß), and hepatocyte growth factor (HGF). Fibrocytes were identified by immunohistochemistry in colonic sections.

**Results:**

CD patients were significantly clustered in a group characterized by increased levels of TH1, TH2 cells, and decreased levels of CD14+ CD163+ monocytes (*p* = .003). In contrast to NSG‐UC mice, NSG‐CD mice exhibited an immune‐remodeling phenotype characterized by enhanced collagen deposition, elevated levels of CD14+ CD163+ monocytes, HGF, and TGFß. This phenotype was further corroborated by the presence of human fibrocytes as components of fibrotic areas.

**Conclusion:**

The NSG‐CD model partially reflects the human disease and allows for studying the development of fibrosis.

## Graphical abstract

Fibrocyte driven fibrosis in the NSG‐CD mouse model.

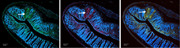

## 1 INTRODUCTION

To date, it is still beyond our understanding to clearly attribute inflammatory pathways to disease‐specific clinical manifestations in chronic inflammatory diseases. This is partially due to the fact that these processes reflect highly dynamic wound healing processes that cover a broad spectrum of immune responses from protection and remodeling to resolving inflammation.^1^, ^2^, ^3^, ^4^ In addition, epithelial cells play a crucial role in these processes and may direct and shape these processes depending on the affected organ.^5^ In the past, the description of inflammatory pathways followed a paradigm that described Crohn's disease (CD) as a TH1^6^, ^7^ and ulcerative colitis (UC) as a TH2‐driven disease.^8^, ^9^ Although both pathways cover crucial processes in the two diseases, it falls short to explain the extensive remodeling of the colon architecture or the monocyte‐driven inflammation observed in UC patients.^10^, ^11^, ^12^ In addition, analysis of cytokine expression of mucosal biopsies revealed no significant differences between the two diseases with exception of interleukin (IL)‐4 and IL‐12p35, both of which were significantly overexpressed in CD as compared to UC patients.^13^ A main difference between the two diseases is that UC is characterized by superficial mucosal inflammation and rectal bleeding and is restricted to the colon,^14^ whereas in CD, inflammation penetrates transmurally, causes perforation, strictures, and extensive fibrosis, and inflammation can affect the entire intestinal tract. Although fibrosis presents a common complication in both diseases, fibrosis follows the inflammatory pattern. In UC, it is restricted to mucosal and submucosal layers, whereas in CD, fibrosis is transmural and causes strictures requiring surgery in up to 80% of patients.^15^, ^16^

Based on the concept that inflammatory processes in the intestine reflect a certain degree of wound healing processes, we recently took on a different approach and developed a panel of immune cells to determine individual profiles of UC patients. This led to the design of a disease map and the identification of subgroups of UC patients characterized by TH1/TH2 versus monocyte‐/TH17‐driven processes.^10^, ^17^ In addition, we developed an animal model that is based on immune‐compromised NOD/Scid IL‐2Rγ^null^ (NSG) mice reconstituted with peripheral blood mononuclear cells (PBMC) from UC patients (NSG‐UC).^18^ In this model, we could show that the pro‐inflammatory and remodeling arm of inflammation acts and can be addressed independently.^17^, ^19^

In this study, we examined whether the combination of patient immune profiling and the examination of the pathological phenotype in NSG mice reconstituted with PBMC from UC (NSG‐UC), CD (NSG‐CD), or non‐IBD (NSG‐non‐IBD) donors differed with regard to the pathological phenotype. The immune profile and the phenotype in the respective mouse models were significantly different. Data indicated that NSD‐CD mice were characterized by intense remodeling of the mucosa.

## 2 MATERIALS AND METHODS

### 2.1 Isolation of PBMC and engraftment

About 60 ml of peripheral blood in trisodium citrate solution (S‐Monovette, Sarstedt) were collected from the arm vein of UC and CD patients as described previously.^20^ The blood was diluted with Hank's balanced salt solution (HBSS, Sigma Aldrich) in a 1:2 ratio. The suspension was loaded onto LeucoSep tubes (Greiner Bio‐One). PBMC were separated by centrifugation at 400*g* for 30 min without acceleration and break. The interphase was extracted and diluted with phosphate‐buffered saline (PBS) to a final volume of 40 ml. Cells were counted and centrifuged at 1400*g* for 5 min. The cell pellet was resuspended in PBS at a concentration of 4 × 10^6^ cells in 100 µl.

Six‐ to eight‐week‐old NOD.cg‐Prkdc^SCID^ Il2rɣ^tm1Wjl^/Szj mice (NSG) were engrafted with 100 µl cell solution into the tail vein on Day 1.

### 2.2 Study protocol

NSG mice were obtained from Charles River Laboratories. Mice were kept under specific pathogen‐free conditions in individually ventilated cages in a facility controlled according to the Federation of Laboratory Animal Science Association (FELASA) guidelines. The protocol was performed as previously described.^19^ Following engraftment on Day 1, mice were presensitized by rectal application of 150 µl of 10% ethanol on Day 8 using a 1 mm catheter (Henry Schein). The catheter was lubricated with lidocaine 2% gel (AstraZeneca). The rectal application was performed under general anesthesia using 4% isoflurane. Post application mice were kept at an angle of 30° to avoid ethanol dripping. On Day 15, mice were challenged by rectal application of 50% ethanol following the protocol of Day 8. On Day 18, mice were sacrificed.

### 2.3 Clinical activity score

The assessment of severity of colitis was performed daily as previously described^10^: Loss of body weight: 0% (0), 0%–5% (1), 5%–10% (2), 10%–15% (3), 15%–20% (4). Stool consistency: formed pellet (0), loose stool or unformed pellet (2), liquid stools (4). Behavior: normal (0), reduced activity (1), apathy (4), and ruffled fur (1). Body posture: Intermediately hunched posture (1) and permanently hunched posture (2). The scores were added daily into a total score with a maximum of 12 points per day. Animals who suffered from weight loss >20%, rectal bleeding, rectal prolapse, self‐isolation, or a severity score >7 were euthanized immediately and not taken into count. All scores were added for statistical analysis.

### 2.4 Macroscopic colon score

The colon was removed, a photograph was taken, and the colon was scored.^10^ Pellet: formed (0), soft (1), liquid (2); length of colon: >10 cm (0), 8–10 cm (1), <8 cm (2); dilation: no (0), minor (1), severe (2); hyperemia: no (0), yes (2); necrosis: no (0), yes (2).

### 2.5 Histopathology

At autopsy, samples from distal parts of the colon were fixed in 4% formaldehyde for 24 h, before storage in 70% ethanol and were routinely embedded in paraffin. Samples were cut into 3 µm sections and stained with hematoxylin and eosin (H&E), periodic acid‐Schiff (PAS), and Masson–Goldner trichrome (MGT, all from Morphisto GmbH).

Epithelial erosions were scored as follows^10^: no lesions (1), focal lesions (2), multifocal lesions (3), and major damage with involvement of basal membrane (4). Inflammation was scored as follows: infiltration of few inflammatory cells into the lamina propria (1), major infiltration of inflammatory cells into the lamina propria (2), confluent infiltration of inflammatory cells into the lamina propria (3), and infiltration of inflammatory cells including tunica muscularis (4). Fibrosis was scored as follows: focal fibrosis (1), multifocal fibrosis and crypt atrophy (2), and general fibrosis and crypt atrophy (3). The presence of edema was scored as follows: focal (1), multifocal (2), and general (3). Hyperemia was scored with one additional point. The scores for each criterion were added into a total score ranging from 0 to 15. Images were taken with an AxioVert 40 CFL camera (Zeiss) using the Zeiss ZE n2 lite software. Figures show representative longitudinal sections in original magnification. In Adobe Photoshop CC, a tonal correction was used to enhance contrasts within the pictures.

### 2.6 Immunohistochemistry (IHC)

For the IHC of colon sections, the samples were fixed in 4% formaldehyde for 24 h, before storage in 70% ethanol, and were embedded in paraffin. Samples were cut into 3 µm sections. Following deparaffinization and rehydration with xylene and ethanol, an antigen retrieval in 1 mM ethylenediaminetetraacetic acid (EDTA) was conducted. Incubation with antibodies followed the previous protocol. Slides were washed twice with PBS and blocking buffer (1% BSA in PBS) was added for 30 min at room temperature. Following the removal of the blocking buffer, the first antibody was diluted in 100 µl blocking buffer (huCD45 1:100, COL1A1 1:100) and incubated overnight at 4°C sealed with parafilm (for antibodies used see Table S2). Following two washing steps with PBS, the second antibody was added at a concentration of 1:400 in 100 µl blocking buffer for 1 h at room temperature. Slides were washed three times with PBS and sealed with cover slides with mounting medium (Anti‐fade gold, Thermo Fisher). Images were taken with an Axiokop 40 CFL camera (Zeiss).

### 2.7 Isolation of human leucocytes

To isolate human leucocytes, spleens were minced and cells filtrated through a 70 µl cell strainer followed by centrifugation at 1400*g* for 5 min and suspended in FACS buffer (1 × PBS, 2 mM EDTA, 2% fetal calf serum [FCS]).^10^ For further purification, cell suspensions were filtrated using a 35 µm cell strainer and then labeled for flow cytometry analysis.

For isolation of lamina propria mononuclear cells (LPMC), a protocol modified by Weigmann et al., 2007 was used.^21^ The washed and minced colon was predigested in an orbital shaker with slow rotation (40 g) at 37°C for 20 min in a predigesting solution containing 1 × ml HBSS (Thermo Scientific), 5 mM EDTA, 5% FCS, 100 U/ml penicillin‐streptomycin (Sigma‐Aldrich Co.). Epithelial cells were removed by filtering through a nylon filter. After washing with RPMI, the remaining colon pieces were digested for 2 × 20 min in a digestion solution containing 1×RPMI (Thermo Fisher Scientific), 10% FCS, 1 mg/ml collagenase A (Sigma‐Aldrich Co.), 10 kU/ml DNase I (Sigma‐Aldrich Co.), and 100 U/ml penicillin‐streptomycin (Sigma‐Aldrich Co.) in an orbital shaker with slow rotation (40*g*) at 37°C.^21^

Isolated LPMC were centrifuged at 1400*g* for 5 min and suspended in 1  × PBS, 2 mM EDTA, 2% FCS (FACS buffer). Cell suspensions were filtrated one more time using a 35 µm cell strainer for further purification before labeling the cells for flow cytometry analysis.

### 2.8 Flow cytometric analysis

Labeling of human leucocytes was performed according to Table S1.

All antibodies (Table S2) were purchased and used according to the manufacturer's instructions (Biolegend). Flow cytometry was performed using an Attune NxT Cytometer (Thermo Fisher Scientific) and analyzed with FlowJo 10.1‐Software (FlowJo LLC).

### 2.9 Detection of cytokines in mouse colon

Approximately 10 mm long sections of the terminal colon were dissected and cleaned of feces with ice‐cold PBS; 350 µl of protease inhibitor cocktail (cOmplete, Roche) was added according to the manufacturer's instructions. Samples were milled for 5 min at 50 Hz with a 5 mm stainless steel bead (Tissuelyser II, Qiagen), centrifuged for 5 min at 300*g*, and 150 µl supernatants were shock frozen and stored at −80°C.

MsCRP (Thermo Fisher Scientific, Cat# EPX01A‐26045‐901, RRID: AB_2575963) and msTGFß (Thermo Fisher Scientific, Cat# EPX01A‐20608‐901, RRID: AB_2575921); msMCP‐3 (Thermo Fisher Scientific, Cat# EPX01A‐26006‐901, RRID: AB_2575933); msHGF was detected by ELISA (Thermo Fisher, Cat# EMHGF) and determined using Tecan infinite 200.

### 2.10 Statistical analysis

Statistical analysis was performed with R: A language and environment for statistical computing. (R Foundation for Statistical Computing. https://www.R-project.org/). Heatmaps were performed using the base package of R (default). Mosaic plots with the VCD package. A Student's *t* test and a 95% confidence interval (CI) were used to compare binary groups and for more than two groups, analysis of variance (ANOVA) followed by the post hoc Tukey's honestly significant difference (TukeyHSD) test was conducted. Variables subjected to ANOVA were tested for normal distribution. All variables fulfilled this requirement. Cumming plots were generated using the dabestr package for data presentation and data comparison. Cumming plots are a new generation of data analysis with bootstrap‐coupled estimation (DABEST) plots that move beyond *p* values.^22^ These plots can be used to visualize large samples and multiple groups easily. When compared to conventional null hypothesis significance testing (NHST) plots, the estimation graphics mainly offer five key advantages: (1) The plot of the full sampling‐error curve of the effect size draws attention to the distribution's graded nature, (2) the difference axis affords transparency to the comparison being made, and (3) in comparison to *p* values, which conflate the magnitude and precision in a single number, the relative size of the CI provides a specific measure of its precision.^22^ (4) The sampling‐error curve is derived with bootstrapping, which makes the method robust and versatile, and (5) the difference diagram encourages the quantitative reasoning about the system under study by focusing on effect size.^22^ Principal component analysis (PCA) was performed in this study to put all in vivo data in context and was generated in R using the plyr, ChemometricsWithR, maptools, car and rgeos packages. Orthogonal partial least square discrimination analysis (oPLS‐DA) was performed using the ropls package.

### 2.11 Ethical considerations

Written informed consent was given by all donors. The study was approved by the Institutional Review Board (IRB) of the Medical Faculty at the University of Munich (2015–2022).

Animal studies were approved by the animal welfare committees of the government of Upper Bavaria, Germany (55.2‐2‐1‐54‐2532‐74‐15) and performed in compliance with German Animal Welfare Laws.

## 3 RESULTS

### 3.1 Comparison of immunological profiles of CD and UC patients

To explore whether CD patients exhibit a similar pattern as observed in UC patients in a previous study,^19^ immune profiles of UC and CD patients were examined by hierarchical clustering analysis using the same panel as in our previous study. Basic patient demographics are presented in Table 1; for the definition of cell types and antibodies used, see Tables S1 and S2; for gating strategy, see Figure S1; and for complete data set, see Table S5.

**Table 1:** Basic patient demographics

	UCN = 47	CDN = 24
Age (years)		
Mean (SD)	38.83 (15.86)	46.9 (18.37)
Range	20–80	21–74
Gender (% male)	55	58
Duration of UC/CD (years)		
Mean (SD)	12.77 (10.08)	15.8 (9.77)
Range	1–39	2–45
SCCAI^23^/CDAI^24^		
Mean (SD)	4.16 (3.44)	50.07 (106.83)
Range	0–12	0–352
Treatment (current)		
TNF‐α‐blocker	17	12
Vedolizumab	15	5
Mesalazine	13	1
Glucocorticoids	5	3
Group		
Aa	17	5
Ab	9	14
B	21	5

**Figure 1:**
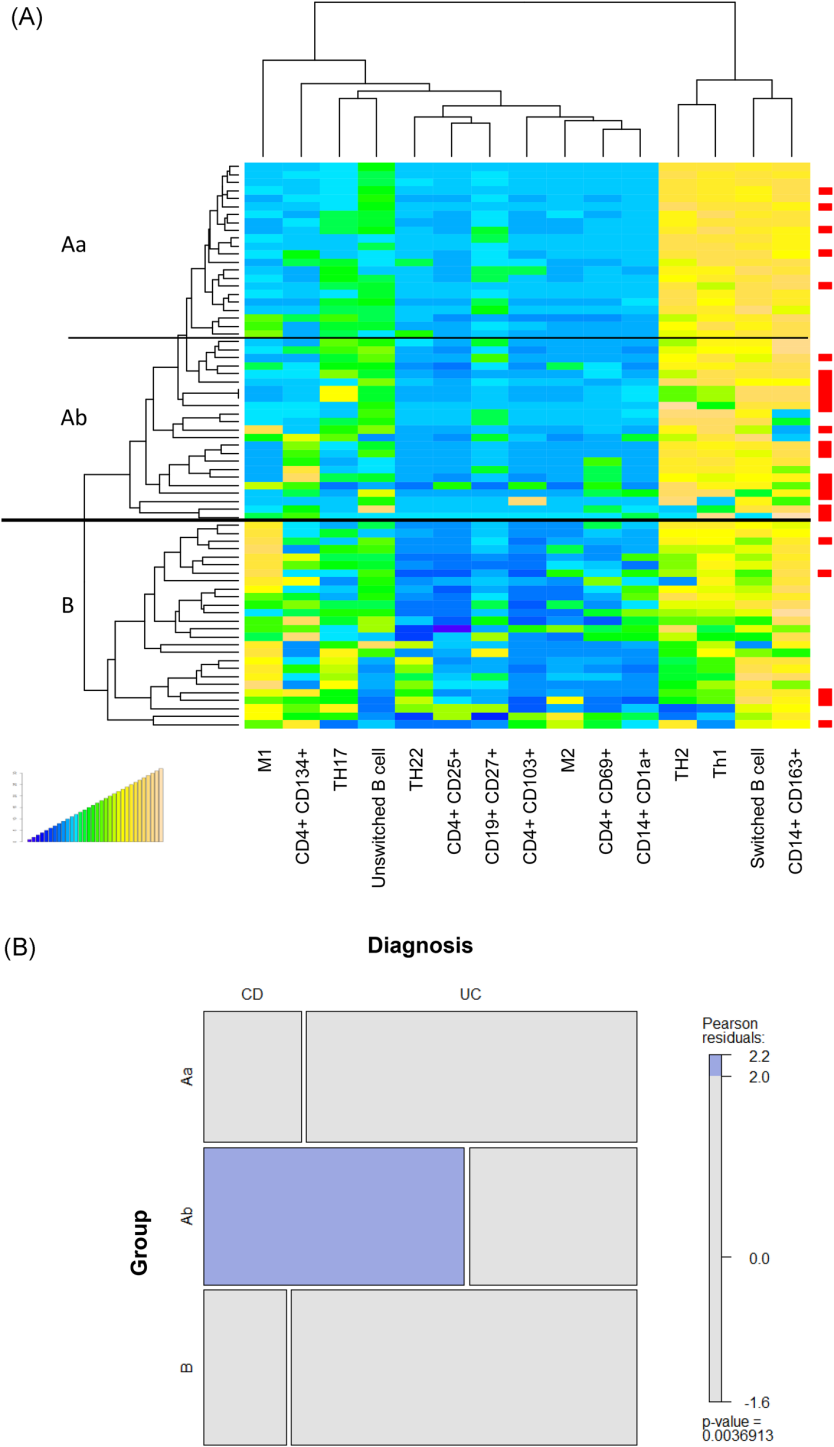
Hierarchical clustering of inflammatory profiles of ulcerative colitis (UC) and Crohn's disease (CD) patients identify subgroups of patients. (A) Frequencies of subtypes of immune cells isolated from blood were analyzed by flow cytometry and depicted as a heatmap (CD: *N* = 24; UC: *N* = 47). Red dots at the right side indicate CD patients. The thick line separates the main Groups A and B, the thin line discriminates subgroups of A (Aa, Ab). The boxes indicate profiles of patients who were selected for reconstitution in the animal study (CD: red; UC: blue). (B) Mosaic plot of subgroups of UC and CD patients with friendly shading. There were more observations of CD patients in Group Ab than would be expected under the null model (*p* = .003)

The panel included subtypes of CD4+ T cells, such as TH1, TH2, TH17, TH22; markers for activated CD4+ T cells like OX40 (CD134), CD69, and CD25; makers for pro‐inflammatory M1 monocytes (CD14+ CD64+ , CD64+ CD1a+) M2 monocytes (CD14+ CD163+, CD14+ CD163+ CD206+); markers for antigen experienced (CD19+ CD27+) and antigen inexperienced (CD19+ CD27−); B cells and switched (CD19+ CD27+ IgD−) and unswitched (CD19+ CD27+ IgD+) B cells. As depicted in the heatmap (Figure 1A) two main Groups (A and B) were identified, which followed the same inflammatory conditions that we have previously observed. Unlike UC patients who were almost evenly distributed between the two groups, CD patients predominantly clustered in Group A signified by TH1, TH2, and switched B cells. In contrast, only five CD patients clustered in Group B characterized by M1 and M2 monocytes, TH17, CD4+ CD69+ and CD4+ CD134+ T cells, and unswitched B cells. Closer inspection revealed that most CD patients clustered in a subgroup of Group A, here defined as Ab. Subgroups Aa, Ab, and B were visualized by a mosaic plot with friendly shading (Figure 1B). Data indicate that the clustering of CD patients in Group Ab is statistically significant (*p* = .003).

**Figure 2:**
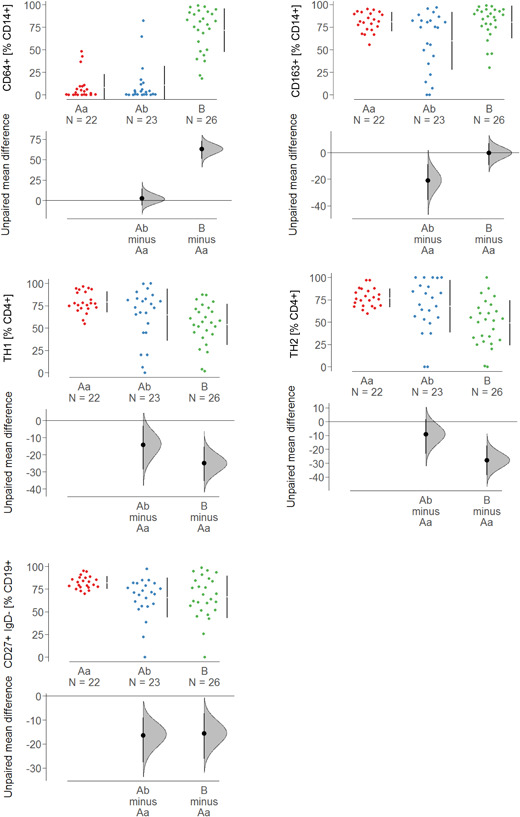
Subtypes of immune cells were differentially expressed in subgroups of Crohn's disease and ulcerative colitis patients. Frequencies of subtypes of immune cells were by flow cytometry and levels depicted as Cumming plots (Aa: *N* = 22; Ab: *N *= 23; B: *N* = 26). The upper part of the plot presents each data point in a swarm plot. The mean and standard deviation (SD) of each group is plotted as a gapped line, where the vertical lines correspond to the mean ± SD and the mean itself is depicted as a gap in the line. In the lower panel of the plots, the effect sizes are shown. The 0 point of the different axis is based on the mean of the reference group (control). The dots show the difference between groups (effect size/mean difference). The shaded curve shows the entire distribution of excepted sampling error for the difference between the means (the higher the peak, the smaller the error). The error bar in the filled circles indicates the 95% confidence interval (bootstrapped) for the difference between means (Aa and Ab refer to the subgroups defined in Figure 1)

To further examine the UC and CD subgroups, quantitative fluorescene activated cell sorting (FACS) analysis of the different immune cells present was performed (Figures 2 and S2). Frequencies of CD4+ CD134+, CD4+ CD25+, CD4+ CD69+, TH1, TH2, TH17, CD14+ CD64+, and CD14+ CD1a were, by way of contrast, elevated in Group B. Both CD64 and CD1a expressing monocytes are considered inflammatory monocytes that have previously been associated with inflammation in UC.^10^, ^25^ In most cases, frequencies of cell types in subgroup Ab followed the pattern of Group Aa with the exception of CD14+ CD163+, CD14+ CCR2+, and CD19+ CD27+ IgD−. These frequencies declined in Group Ab. This observation corroborated the significance of CD14+ CD163+ monocytes contributing to the inflammatory or wound healing process in CD.

### 3.2 Comparison of the NSG‐UC and NSG‐CD mouse model

As we have previously observed that the immunological phenotype of the donor was partially preserved in the NSG‐UC mouse model we speculated that a different pathological phenotype may develop in mice reconstituted with PBMCs from CD patients.^10^ NSG mice were reconstituted with PBMC from UC or CD donors and challenged according to a standard protocol as described in Section Methods, 2. Four of the seven CD patients experienced stricturing complications, four fibrosis (for donor characteristics and numbers of animals in groups see Table 2). The immune profiles of six CD and six UC patients are indicated in Figure 1A. Eight days post reconstitution, the mice were divided into two groups: one was left unchallenged (control) and the other was challenged by rectal application of ethanol (ethanol). Each group contained six to seven animals. In the case of NSG‐UC, the control group contained 12 animals and the ethanol‐challenged group included 37 mice. For the NSG‐CD group, the control group contained 24 mice and the ethanol‐challenged group included 42 animals. For comparison, in two further experiments, mice were also reconstituted with PBMC from non‐IBD donors (control *n* = 8; ethanol *n* = 8).

**Table 2:** Patient characteristics and groups defined in the animal study

						Location						Groups in the NSG model	
Donor	Diagnosis	Group	Medication	Montreal classification	SCCAI/CDAI	**Terminal ileum**	**Colon**	**Ileocolon**	**Upper GI**	Stricturing complications	Fibrosis	Control *n* (f/m)	Ethanol *n* (f/m)
UC 1	UC	B	Infliximab		5								6 (0/6)
UC 2	UC	Aa	Infliximab		6								6 (0/6)
UC 3	UC	Aa	Vedolizumab, glucocorticoids		7							6 (0/6)	6 (0/6)
UC 4	UC	Aa	Vedolizumab glucocorticoids mesalazine		7							6 (4/2)	6 (3/3)
UC 5	UC	B	Vedolizumab, mesalazine		3								7 (5/2)
UC 6	UC	B	Infliximab		12								6 (0/6)
CD 1	CD	B	Vedolizumab	A2/L3/B2	0	x		x		x		6 (6/0)	6 (4/2)
CD 2	CD	Ab	Filgotinib, glucocorticoids	A2/L3/B1	0	x	x	x					6 (5/1)
CD 3	CD	Ab	no	nd	0							6 (0/6)	6 (4/2)
CD 4	CD	B	Vedolizumab, ustekinumab, glucocorticoids	A2/L4/B2	200	x	x	x	x	x	x	6 (2/4)	6 (4/2)
CD 5	CD	B	Glucocorticoids	A2/L3/B2	30	x	x	x		x	x		6 (4/2)
CD 6	CD	B	Entivio	A2/L3/B2	30	x	x	x		x	x		6 (0/6)
CD 7	CD	B	Mesalazine	A2/L3/B2	80	x		x			x	6 (4/2)	6 (2/6)
Non‐IBD 1	Non‐IBD											4 (0/4)	4 (4/0)
Non‐IBD 2	Non‐IBD											4 (4/0)	4 (0/4)

**Figure 3:**
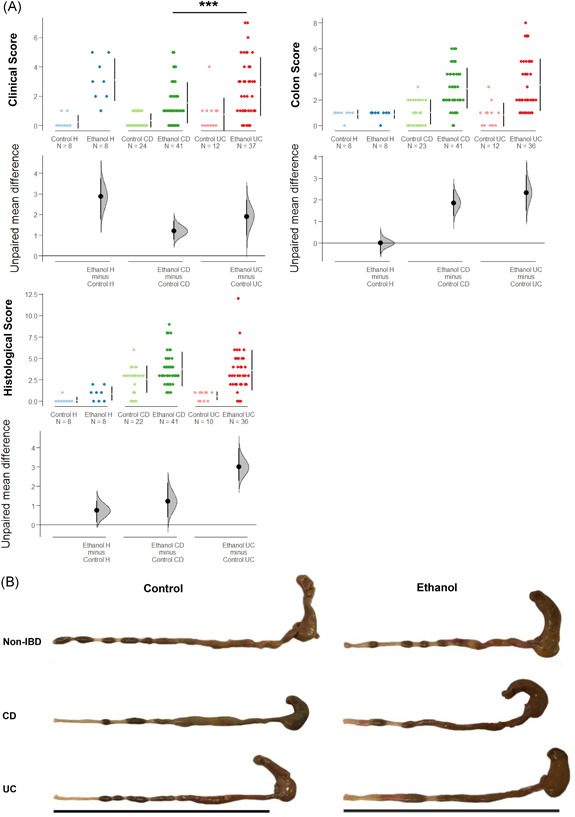
Challenge with ethanol evokes pathological symptoms in NOD/scid IL‐2Rγ^null^ (NSG)‐Crohn's disease (CD) and NSG‐ulcerative colitis (UC) mice. NSG mice were reconstituted with PBMCs from non‐IBD (*N* = 2), CD (*N* = 7), and UC (N = 6) donors and were left unchallenged (control, non‐IBD: *n* = 8; CD: *n* = 24; UC: *n* = 12) or challenged with 10% ethanol at Day 8, and 50% ethanol at Day 15 (ethanol, non‐IBD: *n* = 8; CD: *n* = 41; UC: *n* = 37). (A) Clinical‐, colon‐, and histological scores are depicted as Cumming plots. The upper part of the plot presents each data point in a swarm plot. The mean and standard deviation (SD) of each group is plotted as a gapped line, where the vertical lines correspond to the mean ± SD and the mean itself is depicted as a gap in the line. In the lower panel of the plots, the effect sizes are shown. The 0 point of the different axis is based on the mean of the reference group (control). The dots show the difference between groups (effect size/mean difference). The shaded curve shows the entire distribution of excepted sampling error for the difference between the means (the higher the peak, the smaller the error). The error bar in the filled circles indicates a 95% confidence interval (bootstrapped) for the difference between means. (B) Representative macrophotographs of NSG‐non‐IBD, NSG‐CD, and NSG‐UC colons. Bar corresponds to 10 cm

Upon challenge with ethanol all animals lost weight, and the clinical activity score was reduced. However, the first difference between NSG‐CD and NSG‐UC mice became apparent. Stools became soft or liquid with higher frequency in the UC group as compared to the CD group. In contrast, pellets of control animals appeared normal. Symptoms were classified according to a clinical score described in Section Methods, 2. As shown in Figure 3A, the differences in the clinical scores between the control and the ethanol‐challenged groups were significantly different in the NSG‐non‐IBD and the NSG‐UC mice but not in the NSG‐CD mice (for complete data set see Tables S3 and S4). At autopsy, colons were removed, subjected to macroscopic inspection, and classified according to a colon score described in Section Methods, 2. As shown in Figure 3B, the challenge with ethanol had no effect on the macroscopic appearance of the colons of NSG‐non‐IBD mice. In contrast, colons of ethanol‐challenged NSG‐CD and NSG‐UC mice were markedly different as compared to colons of the respective control mice and the ethanol‐challenged NSG‐non‐IBD mice. Whereas the colons of the latter group appeared normal, pellets of NSG‐CD and NSG‐UC mice became unformed and colons were dilated upon challenge with ethanol. As shown in Figure 3A, the colon score was significantly different in NSG‐UC and NSG‐CD mice when control and ethanol group was compared.

**Figure 4:**
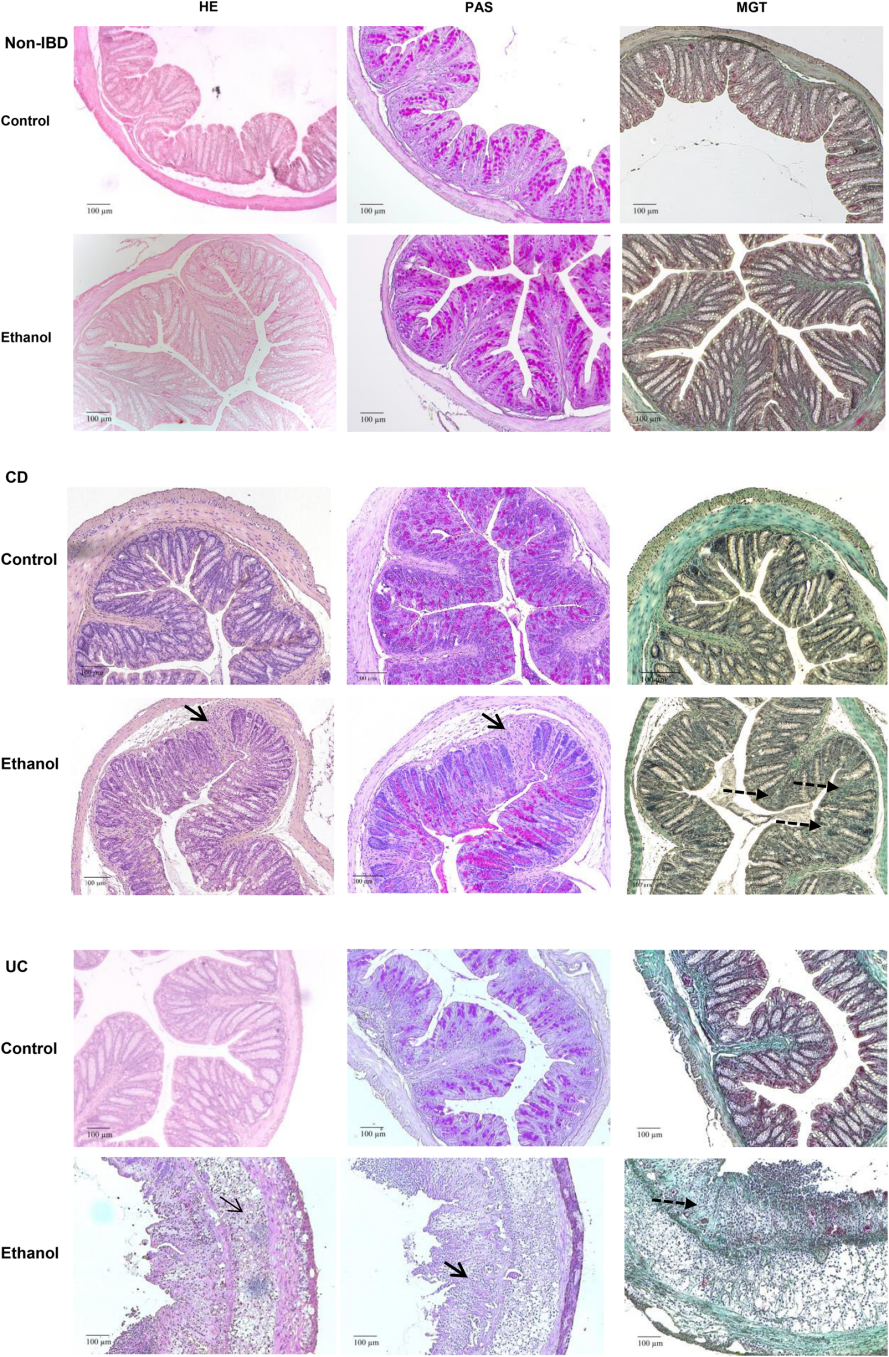
Representative microphotographs of colonic sections from NOD/scid IL‐2Rγ^null^ (NSG)‐non‐IBD, NSG‐Crohn's disease (CD), and NSG‐ulcerative colitis mice reveal differential phenotypes. NSG mice were reconstituted and challenged as described in Figure 3. Arrows indicate edema and influx of inflammatory cells, bold arrows indicate goblet cell loss and dashed line fibrosis. Hematoxylin‐eosin (HE), periodic acid‐Schiff (PAS), Masson–Goldner trichrome (MGT)

For the histological analysis, three stainings were performed: H&E to visualize the alterations in colon architecture, development of edema and influx of inflammatory cells, PAS staining to visualize the loss of goblet cells, and MGT staining to detect fibrosis (Figure 4). This analysis revealed differences between NSG‐CD and NSG‐UC models. The most profound difference was observed when the ethanol‐challenged groups were compared. In the NSG‐non‐IBD group, almost no changes were observed, whereas ethanol had a profound impact in the NSG‐CD and NSG‐UC mice. This impact was, however, different in the latter groups. An influx of inflammatory cells and the development of edema was clearly less significant in the NSG‐CD group. Nevertheless, the challenge with ethanol increased the pathological alterations and evoked loss of goblet cells and fibrosis. In contrast, the influx of a mixed population of inflammatory cells and edema was accompanied by loss of goblet cells and increased collagen deposition in the NSG‐UC mice, whereas the control NSG‐UC group did not spontaneously develop a pathological phenotype. In contrast to both models, this analysis revealed almost nondifference between control and ethanol‐challenged NSG‐non‐IBD mice. Mice were scored according to a histological score as described in Section Methods, 2 (Figure 3A) and the score corroborated the observations. As opposed to NSG‐UC mice, in NSG‐CD mice, the score was already elevated in the absence of ethanol challenge.

**Figure 5:**
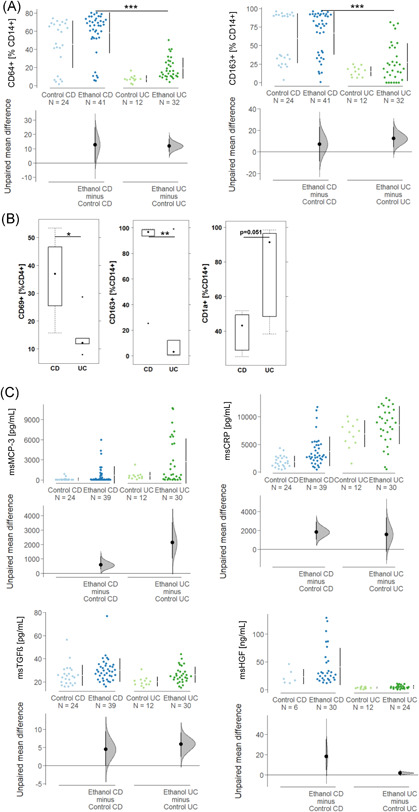
Inflammatory processes are distinct in NOD/scid IL‐2Rγ^null^ (NSG)‐Crohn's disease (CD) and NSG‐ulcerative colitis (CD) mice. Mice were treated as described in Figure 3. (A) Flow cytometric analysis of M1 and M2 monocytes isolated from spleen and their levels depicted as Cumming plots (CD: Control *n* = 24, ethanol *n* = 41; UC: Control *n* = 12, ethanol *n* = 41). (B) Flow cytometric analysis of M1 and M2 monocytes isolated from colon and their levels depicted as boxplots plots. For comparison of groups, a Student's *t* test was conducted. Boxes represent upper and lower quartiles, whiskers represent variability, and outliers are plotted as individual points (0 “***” .001 “**”  .01 “*” .05) (CD:  ethanol *n* = 6 ; UC:   ethanol n = 6). (C) Levels of inflammatory markers (msCRP, msMCP‐3) and remodeling markers (msTGFß, msHGF) in proteins extracted from the colon are depicted as Cumming plots. The upper part of the plot presents each data point in a swarm plot. The mean and standard deviation (SD) of each group is plotted as a gapped line, where the vertical lines correspond to the mean ± SD and the mean itself is depicted as a gap in the line. In the lower panel of the plots, the effect sizes are shown. The 0 point of the difference axis is based on the mean of the reference group (control). The dots show the difference between groups (effect size/mean difference). The shaded curve shows the entire distribution of excepted sampling error for the difference between the means (the higher the peak, the smaller the error). The error bar in the filled circles indicates a 95% confidence interval (bootstrapped) for the difference between means

To further analyze the immunological phenotypes of NSG‐CD and NSG‐UC mice, leukocytes were isolated from the spleen and colon and subjected to flow cytometric analysis. In the spleen, the most pronounced differences were observed when M1 (CD14+ CD64 +) and M2 (CD14+ CD163 +) monocytes were analyzed (Figure 5A). In contrast to NSG‐UC mice, which responded to the challenge with ethanol with increased frequencies of M1 and M2 monocytes, the levels of both of these monocytes were significantly higher in NSG‐CD mice; however, no difference was observed between the control and the ethanol group. The NSG‐non‐IBD mice displayed lower levels of M1 and M2 monocytes and the challenge with ethanol did not evoke significant responses. Analysis of colonic leukocytes corroborated these findings. As numbers of leukocytes in the colon are low in this model, colons of mice were pooled from each group for this analysis. No major difference was observed between control and ethanol‐challenged mice; therefore, only ethanol‐challenged mice were depicted. As observed in leukocytes isolated from the spleen, frequencies of M1 and M2 monocytes were both higher in ethanol‐challenged NSG‐CD mice; however, the difference was only significant for the M2 monocytes (Figure 5B). In contrast, frequencies of CD1a expressing monocytes, which have been suggested to exhibit a pro‐inflammatory phenotype in the NSG‐UC mice,^26^ were significantly elevated. Unlike in NSG‐UC mice, activated CD4+ T cells such as CD4+ CD69+ and CD4+ CD134+ were also elevated in the ethanol‐challenged NSG‐CD mice relative to the ethanol‐challenged NSG‐UC and NSG‐non‐IBD mice; however, the difference was only significant in the case of CD4+ CD69+ T cells. In UC patients, both cell types have been correlated with a monocyte‐driven inflammation.^10^ Finally, mouse neutrophils increased in NSG‐UC colons, further supporting the differences in inflammation in both models. All these analyses corroborated the histological analyses, which suggested a “remodeling inflammation” in NSG‐CD mice.

To examine whether the phenotypic differences between both models were also reflected in the expression of inflammatory markers, proteins were extracted from the colon and subjected to Luminex analysis. As c‐reactive protein (CRP) and the monocyte chemotactic protein (MCP)‐3 have been previously identified as inflammatory markers in the NSG‐UC model,^10^ they were selected to determine potential differences between the NSG‐UC and NSG‐CD model. Indeed, both markers were significantly elevated in both models when ethanol‐challenged groups were compared with the respective control group. However, the levels of CRP were higher in the control and ethanol‐challenged group of NSG‐UC mice as compared to NSG‐CD mice (Figure 5C). This observation further corroborated the more pronounced pro‐inflammatory phenotype of NSG‐UC mice suggested by histological analysis. To further examine the remodeling phenotype of the inflammation in the NSG‐CD model, levels of transforming growth factor‐beta (TGFß) and hepatocyte growth factor (HGF) were determined, which have been identified as significant markers in the NSG‐UC model.^18^ In NSG‐CD mice, TGFß levels were higher in the control and ethanol‐challenged group as compared to NSG‐UC mice, where TGFß levels increased upon challenge with ethanol. In contrast, HGF levels increased significantly in both models upon challenge; however, absolute levels of HGF were significantly higher in NSG‐CD mice. These findings may explain the pronounced crypt elongation in NSG‐CD mice detected by histological analysis. In addition, the elevated TGFß levels also corroborate the observations that the control group in NSG‐CD mice spontaneously developed a pathological “remodeling” phenotype.

**Figure 6:**
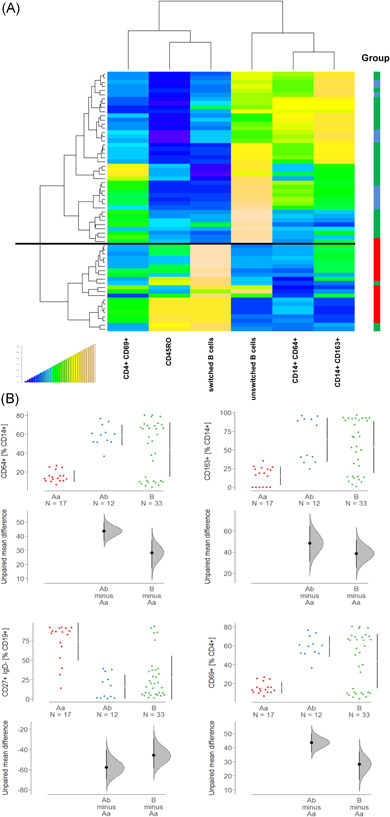
The inflammatory status of the donor is partially conserved in the NSG mouse model. Flow cytometric analysis of human leukocytes isolated from the spleen of NOD/scid IL‐2Rɣ^null^ (NSG) Crohn's disease (CD) and NSG‐ulcerative colitis (UC) mice. Mice were treated as described in Figure 3. (A) Hierarchical clustering. Boxes at the right side indicate group (Aa = red, Ab = blue, B = green). The black line separates the two main groups. (B) Frequencies of leukocytes in groups depicted as Cumming plots. The upper part of the plot presents each data point in a swarm plot. The mean and standard deviation (SD) of each group is plotted as a gapped line, where the vertical lines correspond to the mean ± SD and the mean itself is depicted as a gap in the line. In the lower panel of the plots, the effect sizes are shown. The 0 point of the difference axis is based on the mean of the reference group (Aa). The dots show the difference between groups (effect size/mean difference). The shaded curve shows the entire distribution of excepted sampling error for the difference between the means (the higher the peak, the smaller the error). The error bar in the filled circles indicates a 95% confidence interval (bootstrapped) for the difference between means

To examine whether the inflammatory status of the respective donor is conserved in the mouse models, hierarchical clustering was performed using CD14+ CD64+ (M1), CD14+ CD163+, switched B cells, activated CD4+ T cells (CD4+ CD69+), and antigen‐experienced CD4 T cells (CD45RO+) (Figure 6A). Leukocytes isolated from mice that have been reconstituted with donors from the Aa group clustered in one group, whereas leukocytes derived from donors belonging to the Ab and B groups clustered predominantly in the second group. This pattern corroborated results from previous studies.^19^ Unlike in the analysis of the PBMCs, no distinction was detected between Ab and B groups. This observation was corroborated by the analysis of frequencies in the respective groups (Figure 6B). With the exception of switched B cells, which followed the pattern observed in PBMCs, the frequencies of monocytes were similar to those in the B group.

**Figure 7:**
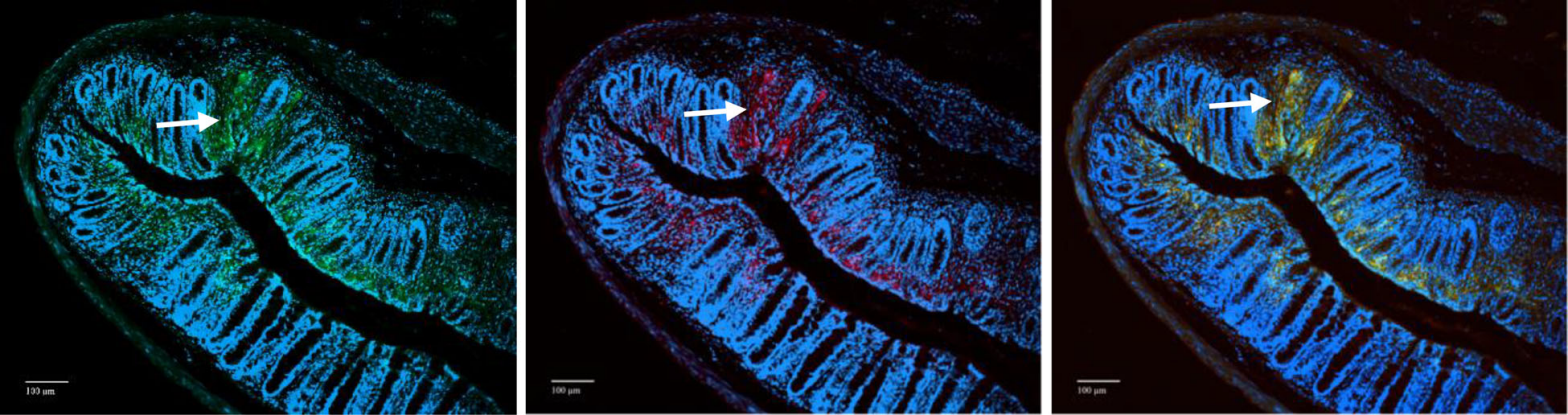
Immunohistochemistry identifies human fibrocytes in fibrotic areas of colonic sections of NOD/scid IL‐2Rγ^null^ Crohn's disease mice. Sections displaying fibrosis were stained with (A) antihuman CD45 (green Alexafluor), (B) anti‐COL1A1 (red Alexafluor). (C) Merged staining

To identify the cells in the fibrotic areas of the colonic sections, immunohistochemical analysis was performed with antihuman CD45 and anti‐COL1A1, which are considered markers for fibrocytes. As shown in Figure 7A, fibrotic areas are infiltrated by human leukocytes and display elevated COL1A1 deposition. The merged picture (Figure 7C) identifies most of the leukocytes as fibrocytes.

## 4 DISCUSSION

One hallmark of Crohn's disease is extensive intestinal fibrosis that is the main cause for therapeutic intervention. To date, most therapeutics, such as anti‐TNF‐alpha monoclonal antibodies, azathioprine, or tofacitinib address the pro‐inflammatory arm of inflammation and neglect the remodeling arm, and only recently, the involvement of fibrocytes, fibroblasts, epithelial cell, and monocytes have come into the focus and gathered interest.^5^, ^11^, ^12^, ^27^, ^28^ Fibrocytes that differentiate from monocytes in the presence of IL‐13 ^29^ and turn into myofibroblasts upon exposure to TGFß ^30^ are one source of collagen I in inflamed tissue.^27^ Fibrocytes participate in both physiological wound healing and pathological fibrosis to include CD.^31^, ^32^

In the study presented here, we have demonstrated that the pathological phenotype of NSG‐CD mice partially reflects the human disease Crohn's disease and is markedly different from the NSG‐UC mice. In contrast to NSG‐UC mice, inflammation in NSG‐CD mice seemed more driven by colon remodeling and less by pro‐inflammatory processes. This hypothesis was supported by histological analysis demonstrating increased collagen deposition, crypt elongation, and less influx of inflammatory cells into the colon in NSG‐CD mice, by the presence of increased frequencies of M2 monocytes in the spleen and colon and by increased levels of TGFß and HGF in the colon as compared to the NSG‐UC mice. Furthermore, IHC identified cells in fibrotic areas as fibrocytes. These observations are in line with clinical manifestations of CD signified by extensive fibrosis. In addition, like in the NSG‐CD model, frequencies of CD14+ CD163+ monocytes were observed to be increased in colonic sections of CD patients.^33^

We observed one important difference between the two mouse models. NSG‐UC mice reconstituted with PBMCs from UC patients who clustered in the monocyte‐driven arm of inflammation also displayed higher levels of M1 monocytes in the mouse model. Most CD patients, however, clustered in the TH1‐/TH2‐driven arm of inflammation, yet NSG‐CD displayed higher levels of M1 and M2 monocytes as compared to those in NSG‐UC mice. Thus, the remodeling arm of inflammation seemed to be induced in this model and did not reflect the apparent pro‐inflammatory profile of the CD patient population. Frequencies of CD14+ CD163+ monocytes were similarly high in subgroups Aa and B, whereas frequencies were relatively lower in group Ab. One possible explanation may be the shedding of the receptor by ADAM 17 metalloproteinase to give rise to the soluble form of sCD163. This mechanism is thought to suppress adaptive immunity.^34^

As previously observed, the mouse models partially reflect the immunological status of the donors. Mice reconstituted with PBMCs from the pro‐inflammatory Aa group were clearly distinct from the B group. However, leukocytes failed to reflect the subtle differences observed in PBMCs. This may be due to the fact that T helper cells were not analyzed. Alternatively, it may indicate the limitations of the mouse model to reflect subtle differences in immunological profiles.

Our findings also reveal a shortcoming of the inflammatory panel selected for clustering patients. This panel has been focused on TH1/TH2 and subtypes of monocytes and has to be expanded to include markers of cytotoxic T cells in future studies as these cells may be a driving force for mucosal destruction.^35^

The same reservation also applies to the NSG‐CD model, which also has to be analyzed for cytotoxicity in the future. In addition, the NSG‐CD model only reflects short‐lived inflammation and, therefore, lacks the capacity to reproduce the inflammation in humans who endure lifelong inflammation in which fibrosis may also be driven by proliferative fibroblasts, epithelial−mesenchymal transition, or endothelial mesenchymal transition.^36^, ^37^, ^38^ A further shortcoming may be the challenge with ethanol that has been used to compare both models but may not necessarily be the right challenge for NSG‐CD mice. Even in light of these limitations, however, the NSG‐CD model presents an improvement as it at least partially reflects the human disease and it can be used to test the efficacy of therapeutics directed against human targets to include monocyte‐derived fibrocytes. In summary, the NSG‐IBD models may become powerful tools to get a better understanding of underlying inflammatory processes in both diseases.

## CONFLICT OF INTERESTS

Anna‐Lena Unterweger, Marietta Seuß, Alena Rüscher, Paula Winkelmann, Roswitha Gropp, and consumables were funded by Shaw Research. The funders had no role in study design, data collection, and analysis, decision to publish, or preparation of the manuscript. Roswitha Gropp has a consulting agreement with D. E. Shaw Research, LLC, New York, USA. D. E. Shaw Research has filed a patent application for DES1 and its use.

## AUTHOR CONTRIBUTIONS

*Ex vivo analysis and the animal studies*: Anna‐Lena Unterweger; *histological analysis*: Anna‐Lena Unterweger and Alena Rüscher and Marietta Seuß; *animal studies and cytokine expression analysis*: Paula Winkelmann; *recruitment of patients, patient history*: Simone Breiteneicher, Florian Beigel, and Leandra Koletzko; *study design and analysis of data*: Matthias Siebeck; *writing of the manuscript, data analysis, study design*: Roswitha Gropp. *writing of the manuscript*:Attila Aszodi.

## Supporting information

Supporting information.

Supporting information.

Supporting information.

Supporting information.

Supporting information.

Supporting information.

Supporting information.

## Data Availability

All data are presented in the manuscript.
