# Supplementary material for: NOD/scid IL‐2Rγnull mice reconstituted with peripheral blood mononuclear cells from patients with Crohn's disease reflect the human pathological phenotype
Source: Immun Inflamm Dis. 2021 Sep 9;9(4):1631–47. doi: 10.1002/iid3.516 (PMC8589348; doi:10.1002/iid3.516)
Supplement: Supplementary file 1 — Supporting information. [file IID3-9-1631-s007.docx]

**A**

CD4+

CD4+ CD134+

CD4+ CD103+

CD4+ CD69+

CD4+ CD25+

CD4+ CD25+ CD127-

CD62L± / CCR7±

CD4 effector memory

CD4 central memory

CD4+ CD45RO+ / RA+

CD4 naive

CD4 effector

CD8 central memory

CD8 effector memory

CD8 effector memory

CD8 naive

CD4+ CCR4± CCR6±

CD4+ CCR4+ CCR6+ CCR10+

CD4+ CXCR3± CCR6±

CD4+ CXCR3+ CCR6±

CD4+ CCR4± CXCR3±

CD4+ RO+ 62L± CCR7±

CD4+ CCR4+ CCR10± CXCR3±

CD4+ CCR4+ CCR6+

CD14+ CD163+ CD206+

CD14+ CD64+

CD14± CD16±

CD14+ CD163+ CD206+

CD14+ TSLPR+

CD14+ CD1a+ CD206+


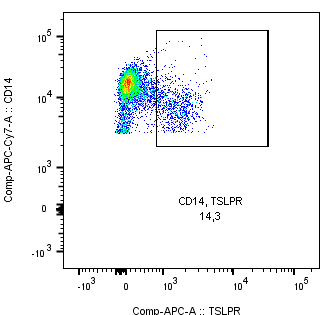

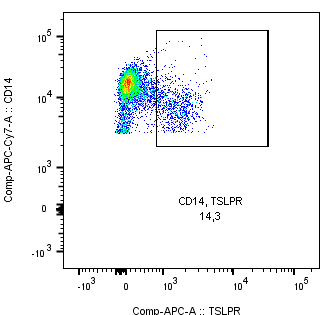

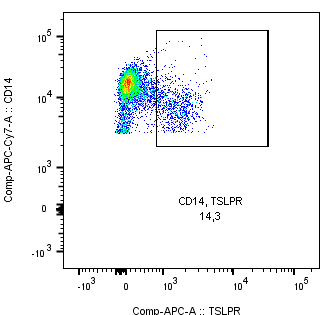

CD19+

CD27±

IgD±

**B**

CD27- IGD±

CD19+ CD27±

CD19+

CD27+ IgD±

CD4+ CD45RO+ CD62L± CCR7±

CD4+ CD45RO+ CD45RA+

CD4+ CD45RA+ CD62L± CCR7±

CD14+ CD64+

CD14+ CD163+

CD14+ CD163+ CD206+ CD206+

CD14+ CD1a+

CD4+ CD25+ CD127 low

CD4+ CD103+

CD4+ CD69+

CD4+ CD134+


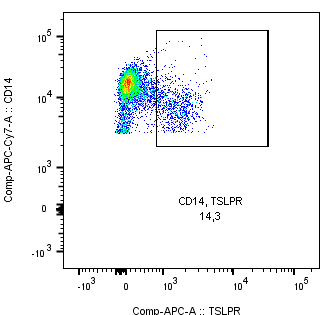


CD4+

CD14+ TSLPR+

**C**

CD14+ CD64+

CD14+ CD163+

CD14+ CD1a+

CD4

CD4+ CD69+

CD4+ CD134+

CD4+ CD103+

msCD11b±

msCD11b+ Ly6G+

huCD15+ CD16+

**Supplementary Figure 1 Gating Strategy.** (A) Human PBMC; (B) Human leukocytes isolated from mouse spleen; (C) Human and mouse leukocytes isolated from mouse colon.

00
