# Supplementary material for: NOD/scid IL‐2Rγnull mice reconstituted with peripheral blood mononuclear cells from patients with Crohn's disease reflect the human pathological phenotype
Source: Immun Inflamm Dis. 2021 Sep 9;9(4):1631–47. doi: 10.1002/iid3.516 (PMC8589348; doi:10.1002/iid3.516)
Supplement: Supplementary file 2 — Supporting information. [file IID3-9-1631-s004.docx]

**

*

***

***

***

*

*

*

**

**

**

**

***

***

***

***

**Aa Ab B**

**Aa Ab B**

**Aa Ab B**

**Aa Ab B**

**Aa Ab B**

**Aa Ab B**

***

*

**Figure S2. Subtypes of immune cells were differentially expressed in subgroups of CD and UC patients.** Frequencies of subtypes of immune cells were analyzed by flow cytometry and levels are depicted as boxplots. For comparison of groups, ANOVA followed by Tukey’s HSD was conducted. Boxes represent upper and lower quartiles, whiskers represent variability and outliers are plotted as individual points (0 '***' 0.001 '**' 0.01 '*' 0.05). Labels given on x-axes on the bottom row apply to all charts.
