# Supplementary material for: NOD/scid IL‐2Rγnull mice reconstituted with peripheral blood mononuclear cells from patients with Crohn's disease reflect the human pathological phenotype
Source: Immun Inflamm Dis. 2021 Sep 9;9(4):1631–47. doi: 10.1002/iid3.516 (PMC8589348; doi:10.1002/iid3.516)
Supplement: Supplementary file 3 — Supporting information. [file IID3-9-1631-s006.docx]

**Supplementary Table S1 Cellular markers used to define immune cells**

| **Marker** | **Definition** |
| --- | --- |
| CD19+ CD27+ IgD+ | Unswitched memory B-cell |
| CD19+ CD27+ IgD- | Switched memory B-cell |
| CD19+ CD38+ | Plasma cell |
|  |  |
| CD4+ CD45RO+ CD62L- CCR7- | Effector memory CD4+ T- cell |
| CD4+ CD45RO+ CD62L+, CCR7+ | Central memory |
| CD4+ CD103+ | Mucosal regulatory CD4+ T cell |
| CD4+ CCR4+ | Th2, effector regulatory T- cell |
| CD4+ CD25+ CD127- | Regulatory T-cell |
|  |  |
| Th1 | CD4+ CXCR3+ |
| Th2 | CD4+ CCR4+ CCR6- |
| Th17 | CD4+ CCR4+ CCR6+ |
| Th22 | CD4+ CCR4+ CCR6+ CCR10+ |
|  |  |
| CD4+ CD134+ | Activated CD4+ T cell |
| CD4+ CD69+ | Activated CD4+ T cell |
| CD4+ CD25+ | Activated CD4+ T cell |
|  |  |
| CD14+ | MC* |
| CD14+ TSLPR+ | MC, expressing TSLPR |
| CD14+ CD64+ | M1MC, FcγR1 expressing |
| CD14+ CD163+ CD206+ | M2 MC, scavenging cells |
| CD14+ CD1a | MC CD1a expressing |
|  |  |
| CD11b+ | cDC1** |
| CD11b+ CD80/86+ | cDC1, mature |
| CD11b+ TSLPR+ | cDC1 TSLPR expressing |
| CD11b+ CD1a+ | cDC1 CD1a expressing |

*Monocyte

******Conventional dendritic cell

TSLPR: Thymic stromal lymphopoietin receptor
