# Supplementary material for: NOD/scid IL‐2Rγnull mice reconstituted with peripheral blood mononuclear cells from patients with Crohn's disease reflect the human pathological phenotype
Source: Immun Inflamm Dis. 2021 Sep 9;9(4):1631–47. doi: 10.1002/iid3.516 (PMC8589348; doi:10.1002/iid3.516)
Supplement: Supplementary file 4 — Supporting information. [file IID3-9-1631-s001.docx]

**Supplementary Table 2. Antibodies used to label human leukocytes**

| **Surfacemarker** | **Colour** | **Cat # RRID #** |
| --- | --- | --- |
| CD19 | Peridine-chlorophyll-protein complex cyanine dye (PerCP-Cy^TM^ 5.5) | **BioLegend Cat# 302230, RRID:AB_2073119** |
| CD38 | Phycoerythrin (PE) | **BioLegend Cat# 356603, RRID:AB_2561899** |
| CD27 | Pe-Cy7 | **BioLegend Cat# 356411, RRID:AB_2562257** |
| IgD | APC/Cy7 | **BioLegend Cat# 348218, RRID:AB_11203722** |
| CD4 | Allophycocyanin (APC)- Cy7 | **BioLegend Cat# 317417, RRID:AB_571946** |
| CD45RA | PE/Cy7 | **BioLegend Cat# 304126, RRID:AB_10708879** |
| CD45RO | PE | **BioLegend Cat# 304206, RRID:AB_314422** |
| CD62L | FITC | **BioLegend Cat# 304838, RRID:AB_2564162** |
| CCR7 | APC | **BioLegend Cat# 353214, RRID:AB_10917387** |
| CXCR3 | FITC | **BioLegend Cat# 353704, RRID:AB_10983066** |
| CCR6 | PE/Cy7 | **BioLegend Cat# 353405, RRID:AB_10918985** |
| CCR10 | APC | **BioLegend Cat# 341505, RRID:AB_2291025** |
| CD8 | PerCP-Cy^TM^ 5.5 | **BioLegend Cat# 344750, RRID:AB_2687201** |
| CD103 | APC | **BioLegend Cat# 350215, RRID:AB_2563906** |
| CD14 | APC-Cy7 | **BioLegend Cat# 325619, RRID:AB_830692** |
| CD16 | PE | **BioLegend Cat# 360703, RRID:AB_2562748** |
| TSLPR | APC | **BioLegend Cat# 322807, RRID:AB_2085327** |
| CD1a (biotin)/secondary Ab streptavidin | FITC | **BioLegend Cat# 300112, RRID:AB_389344** |
| CD64 | PerCP-Cy^TM^ 5.5 | **BioLegend Cat# 305023, RRID:AB_2561585** |
| CD163 | FITC | **BioLegend Cat# 333617, RRID:AB_2563093** |
| CD206 | APC | **BioLegend Cat# 321109, RRID:AB_571884** |
| CD16 | PE | **BioLegend Cat# 302007, RRID:AB_314207** |
| CD11b | APC-Cy7 | **BioLegend Cat# 301341, RRID:AB_2563371** |
| CD11c | PE-Cy7 | **BioLegend Cat# 301607, RRID:AB_389350** |
| CD69 | FITC | **BioLegend Cat# 310903, RRID:AB_314838** |
| CD25 | PE/Cy7 | **BioLegend Cat# 302611, RRID:AB_314281** |
| CD134 (Ox40) | PE | **BioLegend Cat# 350003, RRID:AB_10641708** |
| CD127 | Per CP Cy5 | **BioLegend Cat# 351321, RRID:AB_10900253** |
| **IHC** |  |  |
| Anti-hu CD45 |  | **(BioLegend Cat# 304002, RRID:AB_314390)** |
| Anti COLA1 |  | **Thermo Fisher Scientific Cat# PA5-86949, RRID:AB_2803705** |
| Mouse IgG1 Isotype control |  | **Thermofisher Thermo Fisher Scientific Cat# 14-4714-82, RRID:AB_470111** |
| Rabbit Isotype control |  | **Thermo Fisher Scientific Cat# A-11059, RRID:AB_2534106** |
| Goat anti rabbit | Alexa Fluor 647 | **Thermo Fisher Scientific Cat# A-21244, RRID:AB_2535812** |
| Rabbit anti mouse | Alexa Fluor 488 | **Thermo Fisher Scientific Cat# A-11059, RRID:AB_2534106** |
|  |  |  |
