# Supplementary material for: NOD/scid IL‐2Rγnull mice reconstituted with peripheral blood mononuclear cells from patients with Crohn's disease reflect the human pathological phenotype
Source: Immun Inflamm Dis. 2021 Sep 9;9(4):1631–47. doi: 10.1002/iid3.516 (PMC8589348; doi:10.1002/iid3.516)
Supplement: Supplementary file 5 [file IID3-9-1631-s002.docx]

| SupplementaryTable S4 Complete data set from animal study. Analysis of colon. | | | | | | | | | | | | |
|---|---|---|---|---|---|---|---|---|---|---|---|---|
| | **mean** | | | | **SD** | | | | **n** | | | |
| | **CD** | | **UC** | | **CD** | | **UC** | | **CD** | | **UC** | |
| **Leukocytes from colon [% FoP]** | **Control** | **Ethanol** | **Control** | **Ethanol** | **Control** | **Ethanol** | **Control** | **Ethanol** | **Control** | **Ethanol** | **Control** | **Ethanol** |
| CD4+ | 11,39 | | 6,62 | | 9,71 | | 2,55 | | 3 | | 2 | |
| CD4+ CD69+ | 39,26 | 35,62 | 4,4 | 14,86 | 10,68 | 15,29 | 0,74 | 7,99 | 3 | 5 | 2 | 5 |
| CD4+ CD134+ | 42,96 | 47,72 | 19,05 | 18,3 | 4,1 | 31,6 | 3,88 | 16,34 | 3 | 5 | 2 | 5 |
| CD4+ CD103+ | 13 | 16,13 | 3,69 | 6,31 | 5,76 | 13,87 | 0,38 | 3,23 | 3 | 5 | 2 | 5 |
| CD4+ central memory | 49,23 | 19,84 | 74,15 | 48,98 | 48,85 | 44,36 | 29,34 | 40,43 | 3 | 5 | 2 | 5 |
| CD4+ effector memory | 0,69 | 10,19 | 0,51 | 8,69 | 1,2 | 22,25 | 0,51 | 15,08 | 3 | 5 | 2 | 5 |
| CD4+ naive | 12,96 | 34,24 | 80 | 36,24 | 22,45 | 47,2 | 3,11 | 33,74 | 3 | 5 | 2 | 5 |
| CD14+ | 21,23 | 18,69 | 23,15 | 32,24 | 25,31 | 11,31 | 3,18 | 11,81 | 3 | 4 | 2 | 5 |
| CD14+ CD64+ | 57,28 | 43,88 | 1,15 | 8,13 | 46,52 | 40,02 | 1,83 | 10,1 | 3 | 5 | 2 | 5 |
| CD14+ CD163+ | 69,23 | 82,18 | 0,39 | 8,04 | 52,77 | 31,93 | 0,14 | 8,04 | 3 | 5 | 2 | 5 |
| CD14+ TSLPR+* | 10,84 | 6,96 | 13,97 | 36,32 | 13,24 | 8,58 | 17,28 | 40,22 | 3 | 5 | 2 | 4 |
| CD14+ CD1a+ | 37,5 | 39,66 | 95,2 | 74,64 | 20,32 | 12,05 | 6,36 | 28,85 | 3 | 5 | 2 | 5 |
| CD19+ | 51,38 | 48,83 | 2,43 | 21,038 | 42,7 | 41,4 | 1,94 | 37,28 | 3 | 5 | 2 | 5 |
| ms Neutrophils | 17,39 | 31,69 | 0 | 40,85 | 11,76 | 25,64 | 0 | 22,8 | 3 | 5 | 2 | 4 |

| | Δ | | 95% CI | | Δ | | 95% CI | | Δ | | 95% CI | |
|---|---|---|---|---|---|---|---|---|---|---|---|---|
| | CD | | | | UC | | | | CD /UC | | | |
| Leukocytes from colon [% FoP] | Ethanol / Control | p-value | lower | upper | Ethanol / Control | p-value | lower | upper | Ethanol CD / Ethanol UC | p-value | lower | upper |
| CD4+ | | | | | -6,62 | | | | 20,76 | | | |
| CD4+ CD69+ | -3,64 | | | | 10,46 | | | | 29,42 | 0,03 | 1,92 | 39,64 |
| CD4+ CD134+ | 4,76 | | | | -0,75 | | | | 9,82 | | | |
| CD4+ CD103+ | 3,13 | | | | 2,62 | | | | -29,14 | | | |
| CD4+ central memory | -29,39 | | | | -25,17 | | | | 1,5 | | | |
| CD4+ effector memory | 9,5 | | | | 8,18 | | | | -2 | | | |
| CD4+ naive | 21,28 | | | | -43,76 | | | | -13,55 | | | |
| CD14+ | -2,54 | | | | 9,09 | | | | 35,75 | | | |
| CD14+ CD64+ | -13,4 | | | | 6,98 | | | | 74,14 | | | |
| CD14+ CD163+ | 12,95 | | | | 7,65 | | | | -29,36 | 4E-03 | 35,15 | 113,11 |
| CD14+ TSLPR+* | -3,88 | | | | 22,35 | | | | 27,79 | | | |
| CD14+ CD1a+ | 2,16 | | | | -20,56 | | | | -9,16 | 5,1E-02 | -70,21 | 0,25 |
| CD19+ | -2,55 | | | | 18,608 | | | | 0,00 | | | |
| ms Neutrophils | 14,3 | | | | 40,85 | | | | 0 | | | |
