# Supplementary material for: NOD/scid IL‐2Rγnull mice reconstituted with peripheral blood mononuclear cells from patients with Crohn's disease reflect the human pathological phenotype
Source: Immun Inflamm Dis. 2021 Sep 9;9(4):1631–47. doi: 10.1002/iid3.516 (PMC8589348; doi:10.1002/iid3.516)
Supplement: Supplementary file 6 [file IID3-9-1631-s003.docx]

Table S3 Complete data set from animal study

	Mean						SD						n					
	non-IBD		CD		UC		non-IBD		CD		UC		non-IBD		CD		UC	
Leukocytes from spleen [% FoP]	Control	Ethanol	Control	Ethanol	Control	Ethanol	Control	Ethanol	Control	Ethanol	Control	Ethanol	Control	Ethanol	Control	Ethanol	Control	Ethanol
CD4+	0,9	2,64	16,58	19,1	11,81	16,03	0,56	0,42	8,44	10,75	4,86	16,03	4	3	24	41	12	32
CD4+ CD69+	22,8	40,27	22,09	30,42	22,89	35,55	1,55	15,56	13,03	8,17	10,16	20,88	4	4	24	41	12	32
CD4+ CD134+	71,47	77,65	21,1	23,34	42,2	45,76	3,78	11,57	18,36	17,45	26,56	27,53	4	4	24	41	12	26
CD4+ CD103+	0,28	1,5	13,28	23,34	0,23	1,44	0,06	0,73	12,54	17,45	0,19	1,31	4	4	24	41	12	26
CD4+ central memory	17,75	34,15	15,41	24,84	51,26	34,07	2,57	38,47	19,12	32,66	43,01	33,92	4	4	24	41	12	29
CD4+ effector memory	37,8	39,82	50,4	31,37	33,26	40,01	3,01	24,28	35,89	36,82	29,98	32,19	4	4	24	41	12	29
CD4+ effector	33,12	50,15	16,16	12,92	57,84	47,84	3,45	14,66	25,36	22,95	23,26	38,18	4	4	24	41	12	29
CD4+ naive	5,68	12,91	30,24	48,022	8	23,53	1,69	3,39	38,01	34,48	7,9	33,63	4	4	24	41	12	29
CD8	9,25	2,1	16,67	16,67	2,11	7,41	3,09	2,82	28,52	23,84	2,17	12,74	4	4	12	30	12	29
CD8+ effector memory	16,95	36,4	51,53	46,53	41,25	27,93	2,26	18,15	7,2	26,53	23,83	29,57	4	4	12	30	12	23
CD8+ central memory	9,22	19,61	4,11	3,27	1,61	6,77	3,02	14,56	4,28	7,36	1,32	12,59	4	4	12	30	12	29
CD14+	7	7,89	19,87	31,54	4,7	18,49	3,01	3,53	13,63	22,97	2,9	20,83	4	4	24	40	12	32
CD14+ CD64+	4,96	11,12	45,48	58,15	7,3	19,17	2,37	11,55	26,25	22,49	3,9	11,49	4	4	24	41	12	32
CD14+ CD163+	32,65	32,43	59,62	66,86	15,08	27,5	7,75	9,6	33,54	29,24	6,58	25,42	4	4	24	41	12	32
CD14+ CD163+ CD206+	15,93	17,95	24,18	40,68	4,5	14,32	16,84	17,77	19,77	24,78	3,11	20,76	4	4	24	41	12	26
CD14+ TSLPR+*	13,2	14,46	6,5	5,77	30,53	20,17	8,33	12,25	4,72	4,57	28,18	22,61	4	4	24	41	12	27
CD19+	15,97	10,98	29,67	35,28	9,04	14,12	12,74	7,21	18,54	23,37	2,63	9,61	4	4	24	41	12	35
CD19+ CD27+ IgD+	23,71	38,72	47,58	68,11	53,13	38,7	19,13	25,32	33,22	26,33	44,29	47,81	4	4	24	41	12	35
CD19+ CD27+ IgD-	73,58	56,22	49,29	26,99	44,29	47,81	20,38	26,14	33,12	23,64	38,76	47,81	4	4	24	41	12	35
**Scores**	**Scores**	**Scores**																
Clinical Score	0,25	3,12	0,33	1,53	0,75	2,64	0,46	1,45	0,48	1,39	1,13	2,64	8	8	24	41	12	37
Histological Score	0,12	0,87	2,54	3,75	0,6	3,61	0,35	0,83	1,59	1,99	0,51	2,34	8	8	22	41	10	36
Colon Score	0,75	1	1,04	2,9	0,83	3,16	0,5	0	0,97	1,59	0,93	2,03	8	8	23	41	12	36
**Cytokines [pg/ml]**																		
ms CRP			1883	3758,3	6904,91	8469,01			1024,57	2736,52	2423,66	3420,11			24	40	12	30
ms IL-6			23,77	44,6	5,5	1415,79			5,15	86,83	2,92	3872,25			18	30	8	24
ms MCP-3			102,6	689,37	594,5	2724,23			199,38	1349,15	580,14	3425,72			24	40	12	30
msTGFß			25,62	29,85	28,22	31			8,9	10,11	8,94	10,11			24	40	12	30
msHGF			22122	34321	1501,39	2332,17			7464	18183	820,98	1531,52			12	24	12	24

	Δ		95% CI		Δ		95% CI		Δ		95% CI		Δ		95% CI	
	non-IBD				CD				UC				CD /UC			
**Leukocytes from spleen [% FoP]**	**Ethanol / Control**	**p-value**	**lower**	**upper**	**Ethanol / Control**	**p-value**	**lower**	**upper**	**Ethanol / Control**	**p-value**	**lower**	**upper**	**Ethanol CD / Ethanol UC**	**p-value**	**lower**	**upper**
CD4+	1,74								4,22				-5,13			
CD4+ CD69+	17,47					0,01	-13,96	-1,74	12,66				-22,42			
CD4+ CD134+	6,18								3,56				21,9			
CD4+ CD103+	1,22								1,21				-9,23			
CD4+ central memory	16,4								-17,19				-8,64			
CD4+ effector memory	2,02								6,75				-34,92			
CD4+ effector	17,03								-10				24,492			
CD4+ naive	7,23								15,53	0,02	-29,04	-2,02	18,6			
CD8	-7,15								5,3				-3,5			
CD8+ effector memory	19,45								-13,32				-3,5			
CD8+ central memory	10,39								5,16	0,04	-10	-0,31	13,05			
CD14+	0,89								13,79				38,98			
CD14+ CD64+	6,16								11,87	8E-06	-16,52	-7,14	39,36	7E-11	24,32	44,21
CD14+ CD163+	-0,22								12,42	0,01	-22,28	-2,55	26,36	1E-07	23,58	51,33
CD14+ CD163+ CD206+	2,02				16,5	6E-03	-28,16	-4,8	9,82	0,02	-18,37	-1,12	-14,4	3E-05	14,66	7,20
CD14+ TSLPR+*	1,26								-10,36				21,16	4E-03	-22,69	-4,57
CD19+	-4,99								5,08				29,41			
CD19+ CD27+ IgD+	15,01				20,53	0,002	-41,62	-9,8	-14,43				-20,82	1E-04	19,82	48,56
CD19+ CD27+ IgD-	-17,36				-22,3	0,003	49,29	24,39	3,52				0	2E-03	-38,35	-8,48
**Scores**																
Clinical Score	2,87	6,00E-04	-4,11	-1,63	1,2	5E-06	-1,63	-0,72	1,89	2E-04	-2,8	-0,95	0,14	0,004	-1,99	-0,38
Histological Score					1,21	1E-02	-2,13	0,18	3,01	0E+00	-3,86	-2,15	-0,26			
Colon Score					1,86	2E-06	-2,47	-1,09	2,33	3E-06	-3,2	-1,45	0			
